# Expert opinions on immunotherapy for patients with colorectal cancer

**DOI:** 10.1002/cac2.12095

**Published:** 2020-09-18

**Authors:** Feng Wang, Zi‐Xian Wang, Gong Chen, Hui‐Yan Luo, Dong‐Sheng Zhang, Miao‐Zhen Qiu, De‐Shen Wang, Zhi‐Zhong Pan, Lin Shen, Jin Li, Su‐Zhan Zhang, Rui‐Hua Xu

**Affiliations:** ^1^ Department of Medical Oncology Sun Yat‐sen University Cancer Center, State Key Laboratory of Oncology in South China, Collaborative Innovation Center for Cancer Medicine Guangzhou Guangdong 510060 P. R. China; ^2^ Department of Colorectal Surgery Sun Yat‐sen University Cancer Center State Key Laboratory of Oncology in South China Collaborative Innovation Center for Cancer Medicine Guangzhou 510060 P. R. China; ^3^ Department of Gastrointestinal Medical Oncology Peking University Cancer Hospital Beijing 100142 P. R. China; ^4^ Department of Medical Oncology Tongji University Shanghai East Hospital Shanghai 200120 P. R. China; ^5^ Department of Surgical Oncology The Second Affiliated Hospital of Zhejiang University School of Medicine Hangzhou Zhejiang 310009 P. R. China

## 1 BACKGROUND

With the rapid development of immune checkpoint inhibitors (ICIs) over the past decades, they have become a major area of interest in the treatment of colorectal cancer (CRC) [1, 2]. There are evidence pointing that programmed cell death protein‐1 (PD‐1) blockade, alone or in combination with anti‐cytotoxic T‐lymphocyte‐associated protein 4 (anti‐CTLA4) therapy, achieved durable responses in patients with mismatch repair‐deficient (dMMR) or microsatellite instability‐high (MSI‐H) metastatic CRC (mCRC) [3, 4, 5, 6]. However, the optimal diagnostic method for detecting dMMR/MSI‐H disease as well as the optimal anti‐PD‐1‐based treatment modality still remains controversial in this patient subset. In addition, for the majority of mCRC cases that are mismatch repair‐proficient (pMMR) or microsatellite stable (MSS), the clinical benefits from these agents are generally minimal [3, 7], driving extensive research efforts to develop effective combination therapies in this disease subset. Moreover, investigations of anti‐PD‐1‐based treatments have also been initiated in the non‐metastatic settings of CRC, with some encouraging preliminary evidence [8]. Medical oncologists and surgeons from the Committee of Colorectal Cancer of the Chinese Society of Clinical Oncology had a panel discussion on immunotherapy for patients with colorectal cancer during a seminar on June 16, 2020, in Guangzhou, China. Herein, the expert opinions have been summarized along with relevant clinical evidence (Table 1) to guide real‐world treatment decision‐making regarding the use of ICIs in patients with CRC.

**TABLE 1:** Expert consensus on anti‐programmed cell death protein‐1 (PD‐1) therapy for patients with colorectal cancer

Item	Expert consensus
Approaches to determine MMR or MSI status	IHC should be universally applied to determine MMR status. If available, PCR should also be routinely applied to determine MSI status.
dMMR/MSI‐H mCRC (standard of care)	PD‐1 inhibitors should be adopted as the standard of care first‐line treatment in patients with dMMR/MSI‐H mCRC. PD‐1 inhibitors could be better used in the front‐line than in the second‐ or later‐line settings. Pembrolizumab, nivolumab, and domestic anti‐PD‐1 antibodies approved for some indications by the National Medical Products Administration are considered feasible options.
dMMR/MSI‐H mCRC (special scenarios)	If the treatment goal is rapid control of high‐volume disease, chemotherapy ± targeted therapy is the preferred initial treatment. If the treatment goal is conversion to resectability, anti‐PD‐1 therapy is the preferred initial treatment. For patients with resectable/oligometastatic disease, surgery, and/or ablation in combination with perioperative anti‐PD‐1 therapy is the preferred option. Patients to be treated with PD‐1 inhibitors as part of curative‐intent multimodality therapy should be discussed via a multidisciplinary conference. Anti‐PD‐1 therapy is still the preferred option for patients with an ECOG performance status of 1 and those with *KRAS/NRAS*‐mutated disease.
pMMR/MSS mCRC	Anti‐PD‐1‐based single‐agent or combination therapies are not recommended outside a trial setting.
Non‐metastatic CRC	Patients with non‐metastatic dMMR/MSI‐H CRC should be encouraged to participate in clinical trials on (neo)adjuvant anti‐PD‐1‐based therapies. Anti‐PD‐1‐based single‐agent or combination therapies are not recommended for patients with non‐metastatic pMMR/MSS CRC outside a trial setting.

**Abbreviations**: MMR, mismatch repair; MSI, microsatellite instability; IHC, immunohistochemistry; PCR, polymerase chain reaction; NGS, next‐generation sequencing; CRC, colorectal cancer; mCRC, metastatic colorectal cancer; dMMR, mismatch repair‐deficient; MSI‐H, microsatellite instability‐high; pMMR, mismatch repair‐proficient; MSS, microsatellite stable.

## 2 APPROACHES TO DETERMINE MMR OR MSI STATUS

In the latest consensus (October 23, 2019) from the Committee of Colorectal Cancer of the Chinese Society of Clinical Oncology on the detection of dMMR/MSI‐H disease, three approaches were discussed as potential options: immunohistochemistry (IHC) for MMR proteins, multiplex fluorescent polymerase chain reaction (PCR) for microsatellite sites, and next‐generation sequencing (NGS)‐based MSI algorithms—‐each having its own merits and shortcomings [9]. Moreover, although the concordance between IHC and PCR in examining the MMR/MSI status is generally above 90%, previous evidence suggested that primary resistance of dMMR/MSI‐H mCRC to PD‐1 inhibitors could be largely attributed to a misdiagnosis of the MMR/MSI status and that the majority of the misdiagnosed cases had used only one of the above mentioned diagnostic methods, commonly either IHC or PCR [10]. As such, it was determined to universally use IHC to test the MMR status due to its low cost and high accessibility and to routinely use PCR to test MSI status in institutions with available platforms. In case of discrepant results between IHC and PCR or suspicious primary resistance to anti‐PD‐1 therapy for dMMR/MSI‐H tumors, central review, genetic referral, or a third diagnostic method (e.g., NGS) should be considered.

## 3 ANTI‐PD‐1 THERAPY AS A STANDARD OF CARE FOR UNTREATED dMMR/MSI‐H mCRC

In the recent second interim analysis of the phase III randomized trial, KEYNOTE‐177 [11], pembrolizumab was found to significantly improve the progression‐free survival (PFS), compared to the control group (i.e., doublet chemotherapy ± targeted therapy: either FOLFOX or FOLFIRI, with or without either cetuximab or bevacizumab), as a first‐line treatment for dMMR/MSI‐H mCRC (median PFS, 16.5 vs. 8.2 months; hazard ratio, 0.60; *P* = 0.0002). Superior PFS in patients treated with pembrolizumab, compared to the control group, remained consistent in subgroup analyses of age, sex, race, region, stage, and *BRAF* status. Moreover, a substantially lower rate of grade ≥ 3 adverse events were found in the pembrolizumab arm than in the control arm.

As the overall survival (OS) data from the KEYNOTE‐177 (in the context of a high cross‐over rate) are yet to be released, it remains unclear whether pembrolizumab could be used in second‐ or later‐line settings. However, the tail of the PFS curve with pembrolizumab tended to plateau at the 2‐year PFS rate, reaching 48% [11], suggesting that a substantial proportion of patients might have been “cured” by pembrolizumab as the initial treatment. Moreover, the objective response rate (ORR) with pembrolizumab was numerically higher in the first‐line (KEYNOTE‐177: ORR = 44%) than in the second‐ or later‐line settings (KEYNOTE‐164: ORR = 33%) [4]. Collectively, the panel members agreed that PD‐1 inhibitors should be adopted as the standard of care for first‐line treatment in patients with dMMR/MSI‐H mCRC. Based on existing evidence, PD‐1 inhibitors could be better utilized in the front‐line than in second‐ or later‐line settings.

Compared with pembrolizumab, nivolumab has shown similar efficacy and tolerability profiles in dMMR/MSI‐H mCRC in the single‐arm CheckMate 142 study [5]. Although pembrolizumab and nivolumab are both accessible in China, no anti‐PD‐1 antibodies have been approved for mCRC by the National Medical Products Administration (NMPA, Beijing, China). In this context, pembrolizumab, nivolumab, and domestic anti‐PD‐1 antibodies approved for some indications by the NMPA could be considered as feasible options for patients with dMMR/MSI‐H mCRC. The CheckMate 142 study[5] showed manageable toxicities with first‐line nivolumab + ipilimumab and a numerically higher ORR with this combination than with nivolumab alone (55% vs. 31%)[4]. However, in regard to CTLA4 inhibitors, these are not yet accessible in China, and the safety and efficacy of dual blockade of PD‐1 and CTLA4 require confirmatory investigations before possible recommendations are made.

## 4 INDIVIDUALIZING ANTI‐PD‐1 THERAPY FOR dMMR/MSI‐H mCRC IN REAL‐WORLD PRACTICE

Although PD‐1 inhibitors are likely to become the new standard of care in the first‐line setting for patients with dMMR/MSI‐H mCRC, in real‐world practice, we still need to individualize the use of PD‐1 inhibitors among these patients. Based on data from the KEYNOTE‐177 study and other relevant clinical evidence, expert opinions on preferred treatment options are summarized for the following scenarios:

### 4.1 Rapid control of high‐volume disease as the treatment goal

Although PD‐1 inhibitors exhibit a relatively slow onset of activity in second‐ or later‐line settings (median time to response varying between 2.8 and 4.3 months) [3, 4, 6], the KEYNOTE‐177 study showed comparable median time to response for first‐line pembrolizumab versus chemotherapy ± targeted therapy (2.2 vs. 2.1 months) [11]. Still, PFS curves from the KEYNOTE‐177 study revealed a trend towards shorter PFS among patients treated with pembrolizumab than with chemotherapy ± targeted therapy in the first 6 months after randomization, and the disease control rate was numerically lower among patients treated with pembrolizumab than with chemotherapy ± targeted therapy (65% vs. 75%) [11]. As such, chemotherapy ± targeted therapy could be considered as the preferred initial treatment when the treatment goal is rapid control of high‐volume disease. Based on primary tumor location and RAS/BRAF status, regimens with high ORRs (e.g., doublet chemotherapy + cetuximab for left‐sided, *RAS/BRAF*‐wild type tumors, and triplet chemotherapy + bevacizumab for *RAS* or *BRAF*‐mutant type tumors) are recommended. Moreover, concurrent pembrolizumab in combination with chemotherapy ± targeted therapy should not be routinely administrated in this scenario because of a lack of relevant efficacy data and potentially increased toxicities with the combination treatment. Subsequent anti‐PD‐1 therapy may be considered after achieving disease control by initial chemotherapy ± targeted therapy.

### 4.2 Conversion to resectability as the treatment goal

There are limited data regarding the utility of PD‐1 inhibitors for conversion to resectability, and only around 10% of patients so far in both arms of the KEYNOTE‐177 study have received intent to cure surgery. However, the ORR was numerically higher with pembrolizumab than with chemotherapy ± targeted therapy (44% vs. 33%), and a greater magnitude of radiological tumor regression with pembrolizumab was observed [11]. In addition, pembrolizumab exhibited a better tolerability profile than chemotherapy ± targeted therapy, which is meaningful for patients with a chance of being cured. Therefore, anti‐PD‐1 monotherapy could be considered as the preferred option when the treatment goal is conversion to resectability. The treatment duration using anti‐PD‐1 therapy in this setting should be discussed in a multidisciplinary panel. Moreover, concurrent pembrolizumab in combination with chemotherapy ± targeted therapy should not be routinely administrated in this situation because of a lack of relevant efficacy data and potentially increased toxicities with the combination regimen.

### 4.3 Resectable/oligometastatic disease

Efficacy data from the KEYNOTE‐177 study for pembrolizumab versus chemotherapy ± targeted therapy for patients with resectable/oligometastatic disease are still being awaited. Prior randomized studies failed to demonstrate significant improvements in OS among patients with MSI‐unselected resectable/oligometastatic disease treated with perioperative or adjuvant FOLFOX [12, 13, 14], whereas the value of perioperative or adjuvant chemotherapy remained unclarified in patients with dMMR/MSI‐H resectable/oligometastatic disease. In this context, surgery and/or ablation in combination with perioperative anti‐PD‐1 therapy could be considered as the preferred option, compared with chemotherapy, for patients with dMMR/MSI‐H resectable/oligometastatic disease. The optimal treatment course of pembrolizumab remains undetermined in these patients, which should be discussed in a multidisciplinary panel.

### 4.4 Patients with an Eastern Cooperative Oncology Group (ECOG) performance status of 1 or 2

Prior evidence suggested that patients with impaired performance status could obtain few clinical benefits from anti‐PD‐1 therapies [15, 16]. Similarly, the KEYNOTE‐177 study showed that pembrolizumab failed to demonstrate significant PFS improvement in patients with an ECOG performance status of 1, and those with an ECOG performance status of 2 were excluded [11]. Still, the safety profile was much more favorable with pembrolizumab and the PFS was comparable in the pembrolizumab and control arms in patients with an ECOG performance status of 1 (hazard ratio, 0.84 [95% CI, 0.57‐1.24]). As such, anti‐PD‐1 therapy could be considered as the preferred option for patients with an ECOG performance status of 1. There is a lack of evidence on the utility of anti‐PD‐1 therapy in patients with an ECOG performance status of 2; yet, it might be considered an option when the impaired performance status is a sequel of the tumor.

### 4.5 *RAS/NRAS*‐mutated disease

Subgroup analysis data from the KEYNOTE‐177 study suggested that the efficacy of pembrolizumab could be attenuated in patients with *KRAS/NRAS*‐mutated disease [11]. However, *RAS* status was not available in 30% of the study cohort, and the number of patients with *KRAS/NRAS* mutation was relatively small (*n* = 74), which calls for further confirmatory investigation. Intriguingly, the ORR with nivolumab was numerically lower in patients with *KRAS/NRAS*‐mutated disease than in those with *KRAS/BRAF*‐wild type tumors in the CheckMate 142 study (27% vs. 41%) [5], whereas the KEYNOTE‐164 study [4] showed comparable ORRs with pembrolizumab irrespective of *RAS* status (*RAS*‐mutant type vs. *RAS*‐wild type, 37% vs. 42%). Considering that the safety profile was more favorable with pembrolizumab than with chemotherapy ± targeted therapy and PFS was still comparable in the pembrolizumab and control arms in patients with *KRAS/NRAS*‐mutated disease, anti‐PD‐1 therapy could be considered as the preferred option for this patient subset.

## 5 UTILITY OF ANTI‐PD‐1 THERAPY IN pMMR/MSS mCRC

It is well‐recognized that single‐agent PD‐1 blockade exhibits minimal efficacy in patients with pMMR/MSS mCRC [3, 7]. Current research efforts mainly focus on two strategies to improve the response to anti‐PD‐1 therapy in pMMR/MSS mCRC: the identification of effective biomarkers to predict the efficacy of anti‐PD‐1 therapy, and combination therapies to overcome resistance to PD‐1 blockade.

The US Food and Drug Administration has recently granted accelerated approval to pembrolizumab for the treatment of refractory advanced solid tumors with a high tumor mutational burden (TMB‐H, i.e., TMB ≥ 10 mut/Mb as determined by FoundationOne CDx) based on data from the KEYNOTE‐158 study [17, 18]. One may wonder whether this could be applied to pMMR/MSS mCRC, which was not included in the KEYNOTE‐158 study [17]. Interestingly, a recent single‐arm trial reported an ORR of 11% in mCRC patients with pembrolizumab (25/27 MSS, 2/27 undetermined) with FoundationOne CDx‐based TMB ≥ 9 mut/Mb [19]. Thus far, the Chinese NMPA has not yet approved any approaches for measuring TMB. Moreover, considering the diverse bioinformatics algorithms employed in existing whole‐exome sequencing techniques and NGS panels, it seems unlikely to establish a universal TMB threshold to guide the use of PD‐1 inhibitors in mCRC patients. Altogether, PD‐1 inhibitors are not recommended as a routine use outside a trial setting in patients with pMMR/MSS, TMB‐H mCRC.

Some anti‐PD‐1‐based combination therapeutic regimens (e.g., nivolumab + regorafenib) have shown potential activities in pMMR/MSS mCRC in single‐arm phase I/II studies [20, 21], which require further verification by randomized phase III studies. Thus far, only two relevant phase III studies[22, 23] have released related efficacy data, but unfortunately both have failed to demonstrate improved efficacy of atezolizumab in combination with either chemotherapy + bevacizumab or cobimetinib (a MEK inhibitor). In this context, anti‐PD‐1‐based single‐agent or combination therapies are not recommended as routine use in patients with pMMR/MSS mCRC outside research settings.

## 6 UTILITY OF ANTI‐PD‐1 THERAPY IN NON‐METASTATIC CRC

Existing evidence shows that patients with non‐metastatic dMMR/MSI‐H colon or rectal disease had limited clinical benefits from neoadjuvant FOLFOX (29% of patients showing progression on neoadjuvant FOLFOX) therapy [24, 25], whereas the efficacy of neoadjuvant chemoradiation was comparable among dMMR/MSI‐H and pMMR/MSS rectal cancers (response rate > 90% for both) [25]. In the single‐arm NICHE study[8], neoadjuvant therapy with short‐course nivolumab + ipilimumab exhibited a 100% (20/20) pathologic response rate and 95% (19/20) major pathologic response rate among patients with stage I‐III dMMR colon cancer. Notably, even for those with pMMR disease, this regimen achieved a 27% (4/15) pathologic response rate and 20% (3/15) major pathologic response rate [6]. Moreover, the single‐arm VOLTAGE study[26] has recently shown that neoadjuvant chemoradiotherapy followed by nivolumab could provide a pathologic complete response rate of 60% (3/5) in patients with locally advanced MSI‐H rectal cancer and 30% (11/37) in those with MSS disease.

In the adjuvant setting, 5‐fluorouracil monotherapy might be detrimental to patients with radically resected stage II dMMR/MSI‐H colon cancer [27]. However, the efficacy of adjuvant oxaliplatin seems unaffected by the MMR/MSI status for stage III dMMR/MSI‐H colon cancer [28]. Investigation on the utility of adjuvant anti‐PD‐1 therapy for dMMR/MSI‐H CRC is ongoing (i.e. ClinicalTrials.gov, NCT02912559), and the results are highly anticipated.

## 7 CONCLUSION

Collectively, patients with non‐metastatic dMMR/MSI‐H CRC may be encouraged to participate in clinical trials on (neo)adjuvant anti‐PD‐1‐based therapies. For patients with borderline resectable, locally advanced dMMR/MSI‐H colon cancer, neoadjuvant anti‐PD‐1 therapy (noting that CTLA4 inhibitors are inaccessible in China) could be considered as a reasonable option. Moreover, anti‐PD‐1‐based single‐agent or combination therapies should not be routinely used in patients with non‐metastatic pMMR/MSS CRC outside a trial setting.

## AUTHORS’ CONTRIBUTIONS

Conception and design of the manuscript: XRH, WF, WZX. Manuscript writing: XRH, WF, WZX. Revision of the manuscript: GC, LHY, ZDS, QMZ, WDS, PZZ, SL, LJ, ZSZ. Assembly of data: WF, WZX. All authors read and approved the final manuscript.

## ETHICS APPROVAL AND CONSENT TO PARTICIPATE

Not applicable.

## FUNDING

None to be declared.
